# Human CD8^+^CD28^−^ T Suppressor Cells Expanded by IL-15 *In Vitro* Suppress in an Allospecific and Programmed Cell Death Protein 1-Dependent Manner

**DOI:** 10.3389/fimmu.2018.01442

**Published:** 2018-06-22

**Authors:** Fu Feng, Yanjun Liu, Guihuan Liu, Ping Zhu, Manman Zhu, Hua Zhang, Xiao Lu, Jiumin Liu, Xunrong Luo, Yuming Yu

**Affiliations:** ^1^Department of Urology, Guangdong General Hospital (Guangdong Academy of Medical Sciences), Guangzhou, China; ^2^Department of Immunology, School of Basic Medical Science, Southern Medical University, Guangzhou, China; ^3^Center for Kidney Research and Therapeutics, Northwestern University Feinberg School of Medicine, Chicago, IL, United States

**Keywords:** CD8^+^CD28^−^ T cells, IL-15, suppressor cells, allospecific, programmed cell death protein 1

## Abstract

CD8^+^CD28^−^ T suppressor cells (Ts) have been recently documented to play an important role in alloimmunity. Therefore, understanding and optimizing the conditions under which these cells are generated and/or expanded would greatly facilitate further research and potential clinical use. In this study, we describe rapid expansion of human allospecific CD8^+^CD28^−^ Ts cells through coculture of CD8^+^ T cells with human leukocyte antigen-mismatched donor antigen-presenting cells plus IL-15 in a relative short period of time *in vitro*. Interestingly, IL-15 promotes the expansion of CD8^+^CD28^−^ Ts cells through several parallel mechanisms. The expanded CD8^+^CD28^−^ Ts cells upregulate expression of CD132, CD25, and programmed cell death protein 1 (PD-1), but downregulate expression of CD122, GZM-B, and perforin, while exhibiting no cytotoxicity. Most importantly, the expanded CD8^+^CD28^−^ Ts cells vigorously inhibit CD4^+^ T cells proliferation in a contact-dependent and donor-specific manner both *in vitro* and *in vivo*. Interestingly, the co-inhibitory molecules PD-1 and programmed death-ligand 1 play an obligatory role in the mechanisms of CD8^+^CD28^−^ Ts cells suppression. Taken together, our study report novel methodology for IL-15-induced expansion of human CD8^+^CD28^−^ Ts cells and possible mechanisms. These findings may facilitate understanding of transplant rejection and promote clinical application of CD8^+^CD28^−^ Ts cell-based strategies for inducing and monitoring transplant tolerance in the future.

## Introduction

The indefinite need for non-specific immunosuppressive drugs posttransplantation brings a series of unwanted side effects, such as infectious diseases and malignancies, which often increase the mortality of the patients. Despite of this, a substantial proportion of patients who take immunosuppressive drugs still suffer from chronic rejection that ultimately leads to functional graft loss (1–3). Thus, induction of alloantigen specific tolerance may hold promise for the best therapeutic strategies for transplant patients in the future.

In recent years, CD4^+^ and CD8^+^ regulatory T cell subsets have been found to play an important role in the induction and maintenance of transplant tolerance (4–6). Among them, CD4^+^CD25^+^Foxp3^+^ regulatory T cells (Tregs) are most frequently reported and have been documented to play an important role in various diseases and transplant tolerance (7). A population of predominantly CD8^+^ suppressor T cells has been described in the 1980s (8). To date, several CD8^+^ Tregs population with different markers have been reported. Natural CD8^+^ Tregs marked with CD8^+^CD25^+^, CD8^+^CD122^+^, or CD8^+^CXCR3^+^ (9–11) and induced CD8^+^ Tregs bear various phenotypic characteristics, such as CD28^−^, CD56^+^, CD57^+^, CTLA4^+^, CD103^+^, CD25^+^Foxp3^+^, or LAG3^+^CCL4^+^ (12–15) were reported in different systems.

One of the best characterized CD8^+^ Tregs populations is the CD8^+^CD28^−^ T suppressor cells, which play a significant role in various diseases (16–18) including allogeneic transplantation (19). Human allospecific CD8^+^CD28^−^ T cells have been generated *in vitro* when repeatedly stimulated with allogeneic antigen-presenting cells (APCs) for several rounds and has supplemental recombinant human IL-2 (20). These cells were termed “Ts cells.” Similarly, autoantigen-specific CD8^+^ Tregs including CD8^+^CD28^−^ cells have been induced *in vitro via* stimulation of peripheral T cells obtained from patients with systemic lupus erythematosus and has a supplemental common gamma chain (γc) cytokine cocktail including IL-2, IL-7, and IL-15 (21). Furthermore, IL-15 has also been reported to induce stable loss of CD28 expression in actively dividing CD8^+^CD28^+^ T cells (22, 23). Our previous work has reported a method for rapid expansion of donor-specific human CD8^+^CD28^−^ Ts cells *in vitro* in the presence of cytokines cocktail including IL-2, IL-7, and IL-15, in which IL-15 was found to play a dominant role in *in vitro* expansion of human CD8^+^CD28^−^ T cells (24). Based on the above collective results, we questioned if the conditions for the rapid expansion of donor-specific human CD8^+^CD28^−^ Ts cells in our previous culture system could be optimized, i.e., whether IL-15 alone but cytokines cocktail including IL-2, IL-7, and IL-15 could promote the rapid expansion of donor-specific human CD8^+^CD28^−^ Ts cells *in vitro*. If so, where are these CD8^+^CD28^−^ Ts cells derived from? How about the stability of these cells under *in vivo* conditions? And what might be the mechanisms?

In this study, we cocultured human CD8^+^ T cells and APCs from fully human leukocyte antigen (HLA)-mismatched (HLA-A, -B, and -DR mismatched) volunteers to generate large numbers of CD8^+^CD28^−^ Ts cells with supplemental IL-15 alone instead of cytokines cocktail IL-2, IL-7, and IL-15 *in vitro*. IL-15 promoted the expansion of CD8^+^CD28^−^ Ts cells by several different mechanisms. These IL-15-expanded CD8^+^CD28^−^ Ts cells vigorously suppressed the proliferation of CD4^+^ T cells in an allospecific manner *in vitro*. Most importantly, the allospecific-suppressive capacity of these CD8^+^CD28^−^ Ts cells remained stable when they were adoptively transferred into the NOG mice. Furthermore, programmed death-ligand 1 (PD-1) was upregulated in the expanded CD8^+^CD28^−^ Ts cells, and the blocking of PD-1/PD-L1 signaling in result partially hindered the suppression of these cells. Findings from this study may facilitate the development of new CD8^+^CD28^−^ Ts cell-based strategies for immune tolerance induction, and therapies for prevention of allograft rejection in human organ and tissue transplantation.

## Materials and Methods

### Mice

NOG mice were obtained from Beijing Vital River Laboratory Animal Technology Co. Ltd. (China). Female mice aged 6–8 weeks were used for experiments. All experiments were performed under protocols approved by the Animal Care Committee at the Southern Medical University (Guangzhou, China).

### Human Subjects and HLA Typing

Heparinized peripheral blood samples were acquired from healthy donors with written informed consent. HLA typing was performed by Shenzhen YILIFANG Biotech (CO. LTO. China) using molecular methods. Donors were selected according to their HLA-A, -B, and -DR compatibility or incompatibility based on the specific requirements of individual experiments (Table 1), several groups of donors (designated as A, B, and I), which are fully HLA-A, -B, and -DR mismatched were screened out from 130 volunteers and used for independent experiments.

**Table 1:** Representative human leukocyte antigen (HLA) typing of donors used for experiments.

Cell type	HLA-A		HLA-B		HLA-DR	
A(CD8^+^, CD4^+^, or CD8^+^CD28^−^ T cells)	11	11	37	58 (17)	10	13 (6)
B-antigen-presenting cell (APC)	03	31 (19)	46	51 (5)	04	09
l-APC	02	02	48	48	11 (5)	16 (2)

*Several groups of donors include A, B, and I, which are fully HLA-A, -B, and -DR loci mismatched were screened out from 130 volunteers and used for independent experiments. Table listed is representative of several groups of donors*.

### Peripheral Blood Mononuclear Cells (PBMCs) Isolation and Cell Sorting

Peripheral blood mononuclear cells were isolated from fresh whole blood of healthy donors using lymphocyte separation medium by density gradient centrifugation. CD8^+^ and CD4^+^ T cells were positively selected using CD8 or CD4 isolation kits according to the manufacturer’s instructions (MACS, Miltenyi Biotech). APCs were isolated from PBMCs by depletion of CD2^+^ cells using CD2 Microbeads (Miltenyi Biotech). The purity of resulting population was >95%. When culture of the CD8^+^ T cells for indicated days, CD28^+^ cells were removed by positive selection using human CD28 MicroBead Kit (Miltenyi Biotech, purity > 99%), and the flow-through cells were collected as CD28^−^ cells (purity > 95%). Again, purity of all isolated cells was confirmed by flow cytometry. Isolated CD4^+^ T cells were labeled with 0.5 µM carboxyfluorescein diacetate succinimidyl ester (CFSE) for 7 min at 37°C.

### *In Vitro* Generation and Expansion of CD8^+^CD28^−^ T Cells With Allogeneic APCs and IL-15

2 × 10^6^ purified CD8^+^ T cells from individual A were cultured with 1 × 10^6^ HLA-A, -B, and -DR mismatched APCs from individual B in 2 ml culture medium (RPMI-1640 supplemented with 15% fetal calf serum, FBS, from Gibco-BRL) supplemented with IL-15 (50 ng/ml) (PeproTech Inc., Rocky Hill, NJ, USA) in 24-well plates at 37°C in 5.0% CO_2_ Supplemented culture medium was changed on days 3, 5, and 7 (by replacing 1 ml of the culture medium with fresh medium containing cytokines). Cells in each well were split into two wells on days 5 and 7, and harvested on day 9, and the CD28^−^ population was isolated as described above.

### Suppression of Donor-Specific Proliferation *In Vitro* by Generated CD8^+^CD28^−^ T Cells

5 × 10^4^ CFSE-labeled purified CD4^+^ T cells from individual A (A-CD4^+^ T cells) were used as “responders (R)” and stimulated with 5 × 10^4^ APCs from the original priming donor (individual B; B_-APCs_) or APCs from a HLA-A, -B, -DR fully mismatched indifferent donor (individual I; I_-APCs_), which were used as third party or non-specific stimulation controls. All cultures were prepared in triplicates and incubated in 96-well *U*-bottom plates in 37°C, 5.0% CO_2_ incubator. The CD8^+^CD28^−^ T cells were added as “suppressor (S)” at S:R ratios of 0.5:1. CFSE dilution was assessed on days 7 and 11 to determine the extent of proliferation by flow cytometry.

### The Expanded CD8^+^CD28^−^ T Cells Suppress CD4^+^ T Cells Proliferation *In Vivo*

4 × 10^6^ purified responder CD4^+^ T cells (R) from donor A, 4 × 10^6^ APCs from donor B (B_-APCs_), or from an indifferent donor I (I_-APCs_, HLA-A, B, and DR fully mismatched with donor B and donor A), and 2 × 10^6^ *in vitro* expanded CD8^+^CD28^−^ T cells were added as putative suppressors (S) at S:R ratios of 0.5:1 (with the cell number of “R” kept constant) were adoptively transferred into NOG mice *via* intraperitoneal injection (total volume 1.5 ml). On day 11 after treatment, NOG mice were sacrificed, the spleen was assigned for analysis of human CD4^+^ T cells by flow cytometry or immunohistochemistry.

### Immunohistochemistry

The sections of spleen tissue were dewaxed, rehydrated, and then heated by immersing slides in Tris-EDTA buffer (pH 9.0) for 5 min for antigen retrieval. Subsequently, normal goat serum was used to block non-specific binding and 3% H_2_O_2_ was applied to suppress endogenous peroxidase activity to reduce background staining. The following antibodies were incubated as the manufacturer’s instructions: rabbit anti-human CD8 Ab (ab93278, abcam) and mouse anti-human CD4 (T Helper/Inducer) monoclonal antibody (mAb) (ZM-0418, ZSGB-BIO) in a humidified chamber overnight at 4°C. After thoroughly washing the corresponding slides for 30 min, horseradish peroxidase labeled-goat anti rabbit IgG Ab and goat anti mouse IgG Ab were added. Finally, staining of the tissue sections was performed with an enhanced HRP-DAB chromogenic substrate kit. The sections were counterstained with immunohistochemical staining and visualized under a light microscope (Nikon, Japan).

### Transwell Experiments

The lower chambers of 96-well transwell plates were plated with either 5 × 10^4^ CFSE-labeled CD4^+^ T cells from individual A (A-CD4^+^ T cells), or with A-CD4^+^ T cells and 5 × 10^4^ priming APCs from individual B (B_-APCs_) in the presence and absence of 2.5 × 10^4^ CD8^+^CD28^−^ T cells (total volume 235 µl). The upper chambers were plated with medium, CD8^+^CD28^−^ T cells, or CD8^+^CD28^−^ T cells plus priming APCs (B_-APCs_). Cells collected from the lower chamber after 7 days of culture were assessed by FACS for CFSE dilution.

### Cytotoxic Assay of CD8^+^CD28^−^ T Cells

Carboxyfluorescein diacetate succinimidyl ester-based cytotoxic assay was set up according to published methods (24) as follows. APCs serving as target cells were labeled with two different concentrations of CFSE: high concentration (2.0 mM) for APCs from the priming donor B (B_-APC-_CFSE^high^) and low concentration (0.2 mM) for APCs from an HLA -A, -B, -DR fully mismatched indifferent donor I (I_-APC-_CFSE^low^). The *in vitro* generated CD8^+^CD28^−^ T cells or CD8^+^CD28^+^ T cells were used as putative effector cells. 5 × 10^4^ effector cells were cultured with 5 × 10^4^ B_-APC-_CFSE^high^ and 5 × 10^4^ I_-APC-_CFSE^low^ together in 96-well *U*-bottom plates in triplicate wells. Cultured B_-APC-_CFSE^high^ and I_-APC-_CFSE^low^ in the absence of effector cells were used as controls for spontaneous cytolysis. B_-APC-_CFSE^high^ and I_-APC-_CFSE^low^ were enumerated by FACS after 0, 24, 72, and 120 h of culture. The ratios of cell numbers of I_-APC-_CFSE^low^ vs B_-APC-_CFSE^high^ in the absence or presence of effector cells were calculated over time to estimate specific killing of B_-APC-_CFSE^high^.

### Flow Cytometric Analyses

All anti-human fluorescent monoclonal antibodies were used at concentrations recommended by the manufacturer and according to manufacturer’s instructions. The following anti-human Abs were used: CD4-APC (SK3), CD28-APC (CD28.2), CD8-Percp-Cy5.5 (RPA-T8), CD2-FITC (RPA-2.10), CD56-FITC (MEM188), CD57-FITC (TB01), CD122-FITC (TU27), CD279 (PD-1)-FITC(MIH4), Perforin-FITC (dG9), CD25-PE (BC96), CD178 (FasL)-PE (NOK-1), CD215 (IL-15α)-PE (eBioJM7A4), Granzyme B-PE (GB11), all from eBioscience. CD132-PE (TUGh4, Biolegend). Appropriate isotype control Abs were used: mouse IgG1 κ-APC (P3.6.2.8.1), mouse IgG1 κ-FITC (P3.6.2.8.1), mouse IgM-FITC (11E10), mouse IgG2a κ-FITC (eBM2a), mouse IgG2b κ-FITC (eBMG2b), mouse IgG1 κ-PE (P3.6.2.8.1), mouse IgG2b-PE (eBMG2b). Data acquisition was performed on an LSR Fortessa flow cytometer (BD Bioscience) and analyzed using FlowJov X.0.7 software.

### Statistical Analysis

The results were given as means ± SD of independent experiments. *p*-Values were calculated using SPSS v. 22.0 software with unpaired, two-tailed Student’s *t*-test or where indicated with one-way analysis of variance followed by Turkey’s test. *p*-Values of less than 0.05 were considered to indicate statistical significance. *p < 0.05, **p < 0.01, and ***p < 0.001.

## Results

### IL-15 Promotes Rapid Expansion of CD8^+^CD28^−^ T Cells When Cultured With Allogeneic APCs *In Vitro*

**Figure 1:**
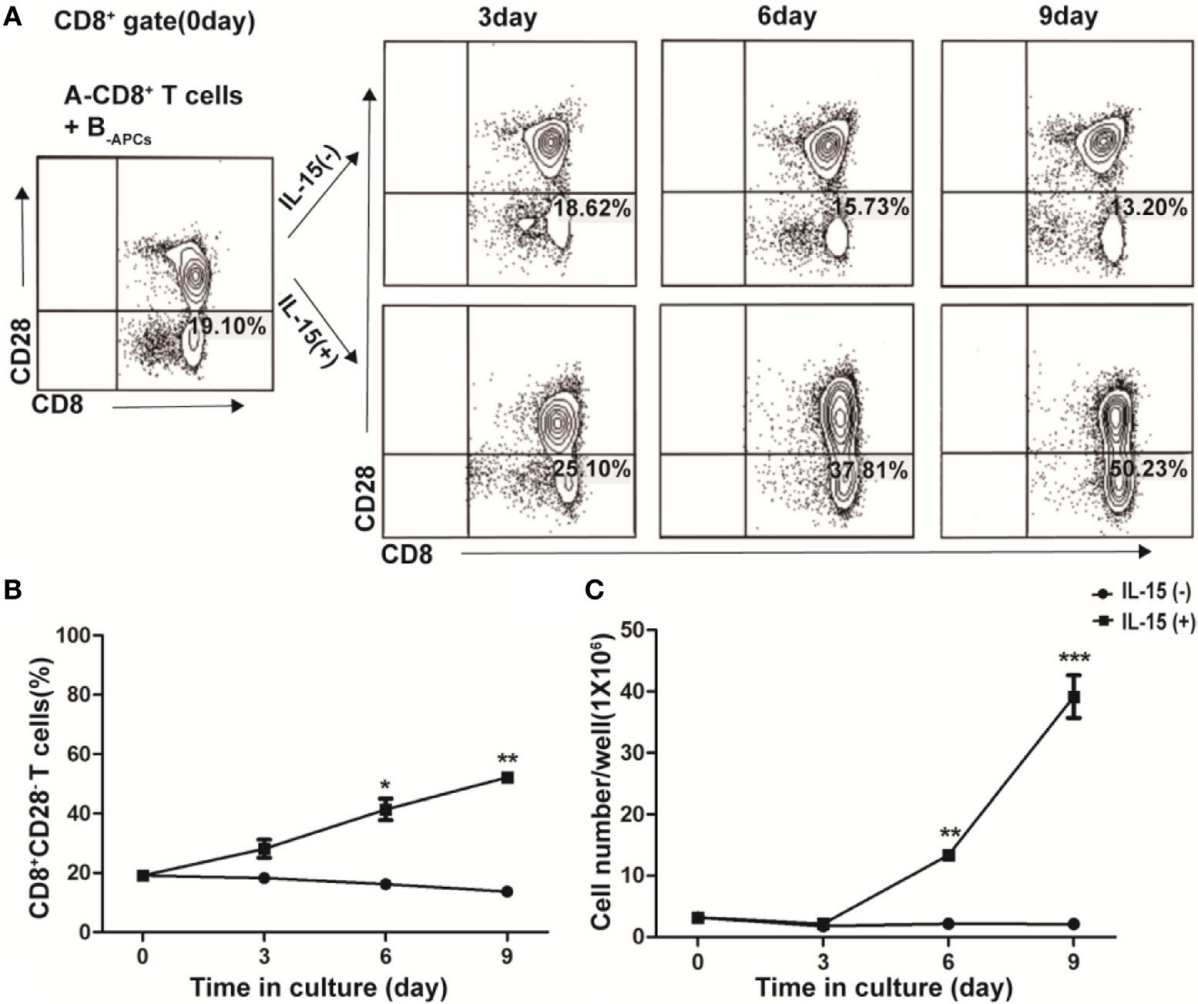
IL-15 promotes rapid expansion of CD8^+^CD28^−^ T cells when cultured with allogeneic antigen-presenting cells (APCs) *in vitro*. CD8^+^ T cells isolated from healthy donor A were stimulated with human leukocyte antigen-A, -B, and -DR fully mismatched APCs from donor B (B-_APC_ cells) in the presence or absence of IL-15 for 9 days in 24-well plates. **(A)** The fraction of CD28^−^CD8^+^ T cells in CD8^+^ T cells were calculated after cultured for 0, 3, 6, and 9 days, respectively. **(B)** The dynamic average fraction of CD8^+^CD28^−^ T cells in CD8^+^ T cells over time. **(C)** Cell number of each well after expansion over time in the presence or absence of IL-15. Data shown in **(A)** was representative of three independent experiments. Data shown in **(B,C)** were the average value of three independent experiments. *p < 0.05, **p < 0.01, and ***p < 0.001.

In freshly isolated human CD8^+^ T cells from PBMCs, CD28^−^ T cells accounted for a small fraction (19.10%, Figure 1A, Left panel). Fresh CD8^+^ T cells from donor A (A-CD8^+^ T cells) were stimulated with HLA-A, -B, and -DR fully mismatched APCs from donor B (B-_APCs_) in the presence or absence of IL-15. The fraction of CD28^−^ T cells within CD8^+^ T cells did not increase in the absence of IL-15, which were 18.62% on day 3, 15.73% on day 6, and 13.20% on day 9, respectively (Figure 1A, upper panel). In contrast, in the presence of IL-15, the fraction of CD28^−^ T cells within CD8^+^ T cells increased dramatically, which accounted for 25.10% on day 3, 37.81% on day 6, and 50.23% on day 9, respectively (Figure 1A, lower panel). Thus, IL-15 could increase the percentage of CD8^+^CD28^−^ T cells in the culture (day 6, *p* = 0.02, and day 9, *p* = 0.003, respectively; Figure 1B). Most importantly, the total number of CD8^+^CD28^−^ T cells increased by 23.42 ± 5.43-fold after 9 days culture in the presence of IL-15 (*p* < 0.001), whereas this increase did not occur in the absence of IL-15 (Figure 1C). Therefore, IL-15 promoted rapid expansion of CD8^+^CD28^−^ T cells when cultured with allogeneic APCs *in vitro*.

### IL-15 Promotes Expansion of CD8^+^CD28^−^ T Cells *In Vitro* Through Different Mechanisms

**Figure 2:**
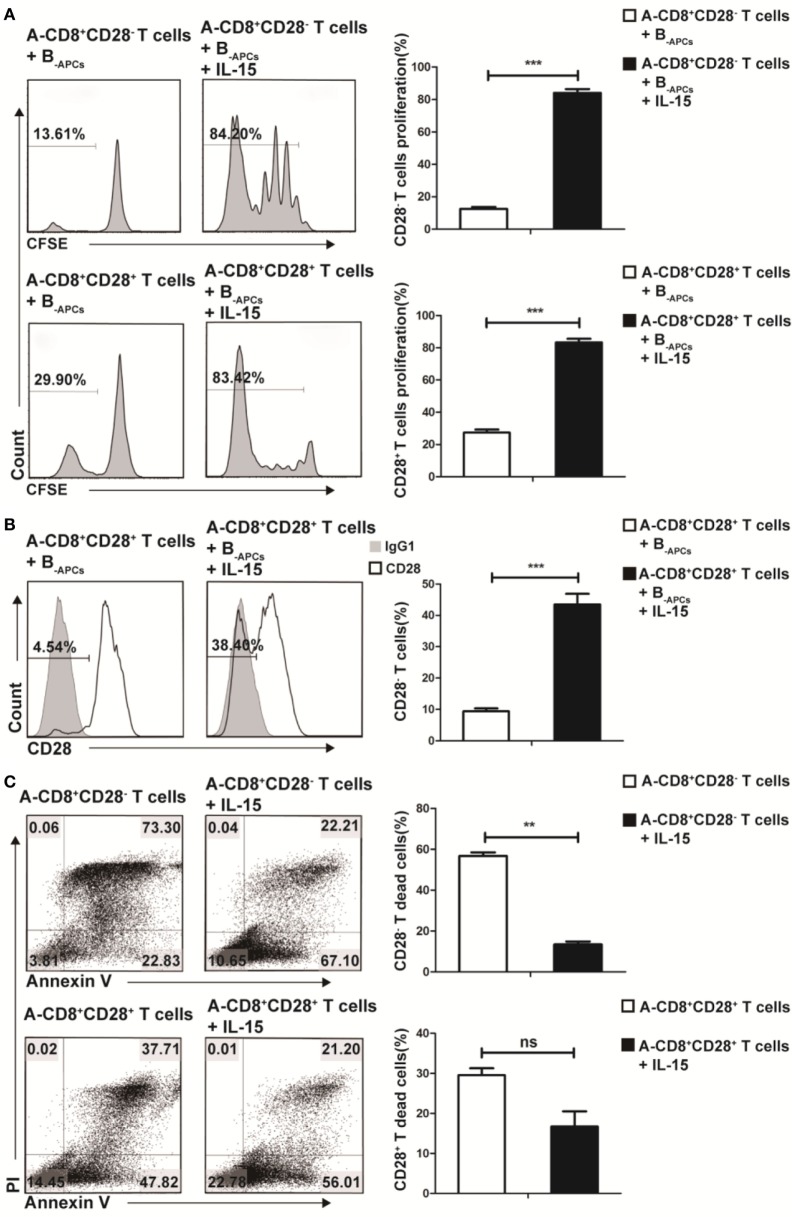
IL-15 promotes expansion of CD8^+^CD28^−^ T cells *in vitro* through different mechanism. Freshly isolated CD8^+^CD28^−^ T cells or CD8^+^CD28^+^ T cells were labeled with carboxyfluorescein diacetate succinimidyl ester and cultured with B_-APCs_ in the presence or absence of IL-15 for 9 days. **(A)** The percentages of proliferating cells in CD8^+^CD28^−^ or CD8^+^CD28^+^ T cells after culture. **(B)** The percentages of CD8^+^CD28^−^ T cells number in CD8^+^CD28^+^ T cells after cultured in the presence or absence of IL-15 for 9 days. **(C)** Apoptosis of CD8^+^CD28^+^ T cells or CD8^+^CD28^−^ T cells when cultured in the presence or absence of IL-15 for 9 days. The bar graphs indicate the means ± SD, *n* = 3. *p < 0.05, **p < 0.01, and ***p < 0.001.

To further understand how IL-15 promoted the expansion of CD8^+^CD28^−^ T cells and where these CD8^+^CD28^−^ T cells derived from, we cocultured purified CD8^+^CD28^−^ or CD8^+^CD28^+^ T cells from donor A with B_-APCs_ in the presence or absence of IL-15 for 9 days. As shown in Figure 2A, in the presence of IL-15, the proliferating fraction of CD8^+^CD28^−^ and CD8^+^CD28^+^ T cells both increased dramatically (from 13.61 to 84.20% for CD8^+^CD28^−^ T cells vs from 29.90 to 83.42% for CD8^+^CD28^+^ T cells). Moreover, the total number of both cell populations significantly increased (*p* < 0.001, both). Secondl we determined the dynamic expression of CD28 on CD8^+^CD28^+^ T cells (A-CD8^+^CD28^+^ T cells) when cultured with B_-APCs_ in presence or absence of IL-15. As shown in Figure 2B, IL-15 accelerated the loss of CD28 expression on CD8^+^CD28^+^ T cells, resulting in an increase of CD8^+^CD28^−^ T cells post-culture (from 4.54 to 38.40%, *p* = 0.001). Finally, as shown in Figure 2C, we examined the influence of IL-15 on the apoptosis of the CD8^+^CD28^−^ and CD8^+^CD28^+^ T cells. For CD8^+^CD28^−^ T cells, after 9 days culture, IL-15 sharply decreased the percentage and total number of dead cells (from 73.30 to 22.21%) (*p* = 0.003). Similarly, IL-15 also decreased the percentage of dead cells among CD8^+^CD28^+^ T cells following coculture (from 37.71 to 21.20%), although the difference in the total number of dead cells did not reach statistical significance (*p* = 0.92). Taken together, these findings indicated that IL-15 promotes the expansion of CD8^+^CD28^−^ T cells by promoting the proliferation of CD8^+^CD28^−^ T cells, accelerating the loss of CD28 on CD8^+^CD28^+^ T cells, and decreasing apoptosis of proliferating CD8^+^CD28^−^ T cells.

### CD8^+^CD28^−^ T Cells Expanded by IL-15 Inhibit Allospecific CD4^+^ T Cell Proliferation *In Vitro*

**Figure 3:**
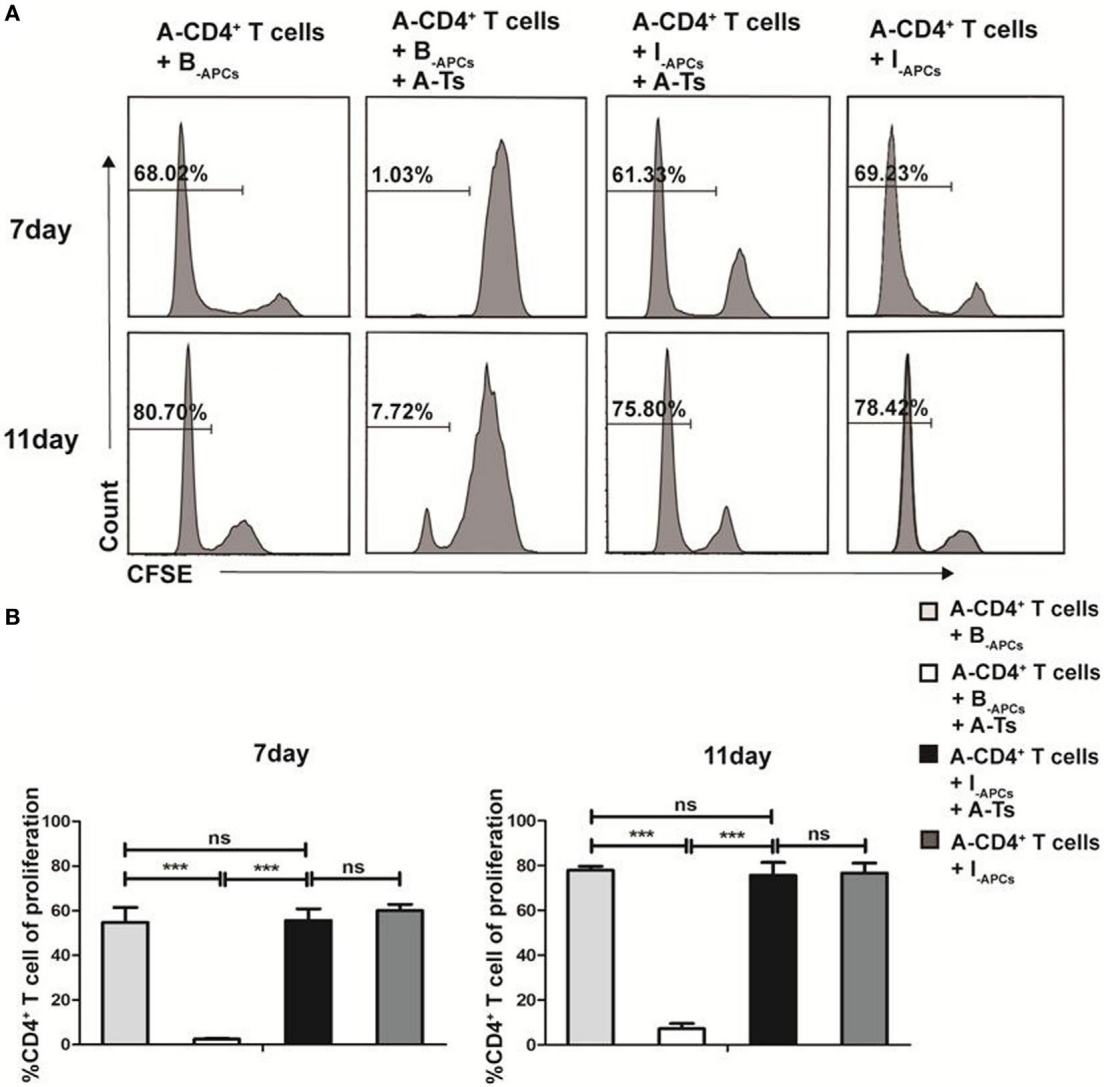
CD8^+^CD28^−^ T cells expanded by IL-15 inhibit the proliferation of CD4^+^ T cells in an allospecific manner *in vitro*. 5 × 10^4^ carboxyfluorescein diacetate succinimidyl ester (CFSE)-labeled responder CD4^+^ T cells (R) from donor A were stimulated with 5 × 10^4^ antigen-presenting cells (APCs) from donor B (B_-APCs_) or indifferent donor I [I_-APCs_, human leukocyte antigen (HLA)-A, -B, and -DR fully mismatched with donor B and donor A] in the presence of absence of 5 × 10^4^ CD8^+^CD28^−^ T cells (A-Ts) in triplicates in 96-well plates for 7 and 11 days. **(A)** The proliferation of CD4^+^ T cells was measured by CFSE dilution. **(B)** The percentages of proliferating CD4^+^ T cells were compared. The bar graphs indicate the means ± SD, *n* = 3. *p < 0.05, **p < 0.01, and ***p < 0.001.

CD8^+^CD28^−^ T cells expanded by IL-15 (designated as A-Ts) were generated from CD8^+^ T cells from donor A by culturing with allogeneic APCs from donor B (designated as B_-APC_ cells) for 9 days in the presence IL-15 as described above. A-Ts were used as suppressors (S) to test their immunosuppressive function in mixed lymphocyte reactions (MLRs) by using CFSE-labeled CD4^+^ T cells isolated from donor A as responder (A-CD4^+^ T cells) and APCs from donor B as stimulator (B_-APCs_). Third party allogeneic APCs from donor I (designated as I_-APCs_), whose HLA-A, B, and DR were fully mismatched with donor A and B (Table 1), were used as control APCs. Suppressor to responder ratio (S:R) was 0.5:1. As show in Figure 3A, proliferation of CD4^+^ T cells on days 7 and 11 were profoundly inhibited when A-Ts were added as suppressor in the culture system with B_-APCs_ stimulator (day 7, 1.03 vs 68.02%, *p* < 0.001; day 11, 7.72 vs 80.70%, *p* < 0.001). When the stimulator was changed from B_-APCs_ to I_-APCs_, A-Ts were not able to inhibit the proliferation of CD4^+^ T cells (Figures 3A,B). Taken together, our results indicated that CD8^+^CD28^−^ T cells driven by IL-15 *in vitro* suppress the proliferation of CD4^+^ T cells in an allospecific manner.

### CD8^+^CD28^−^ T Cells Expanded by IL-15 Inhibit Allospecific CD4^+^ T Cell Proliferation *In Vivo*

**Figure 4:**
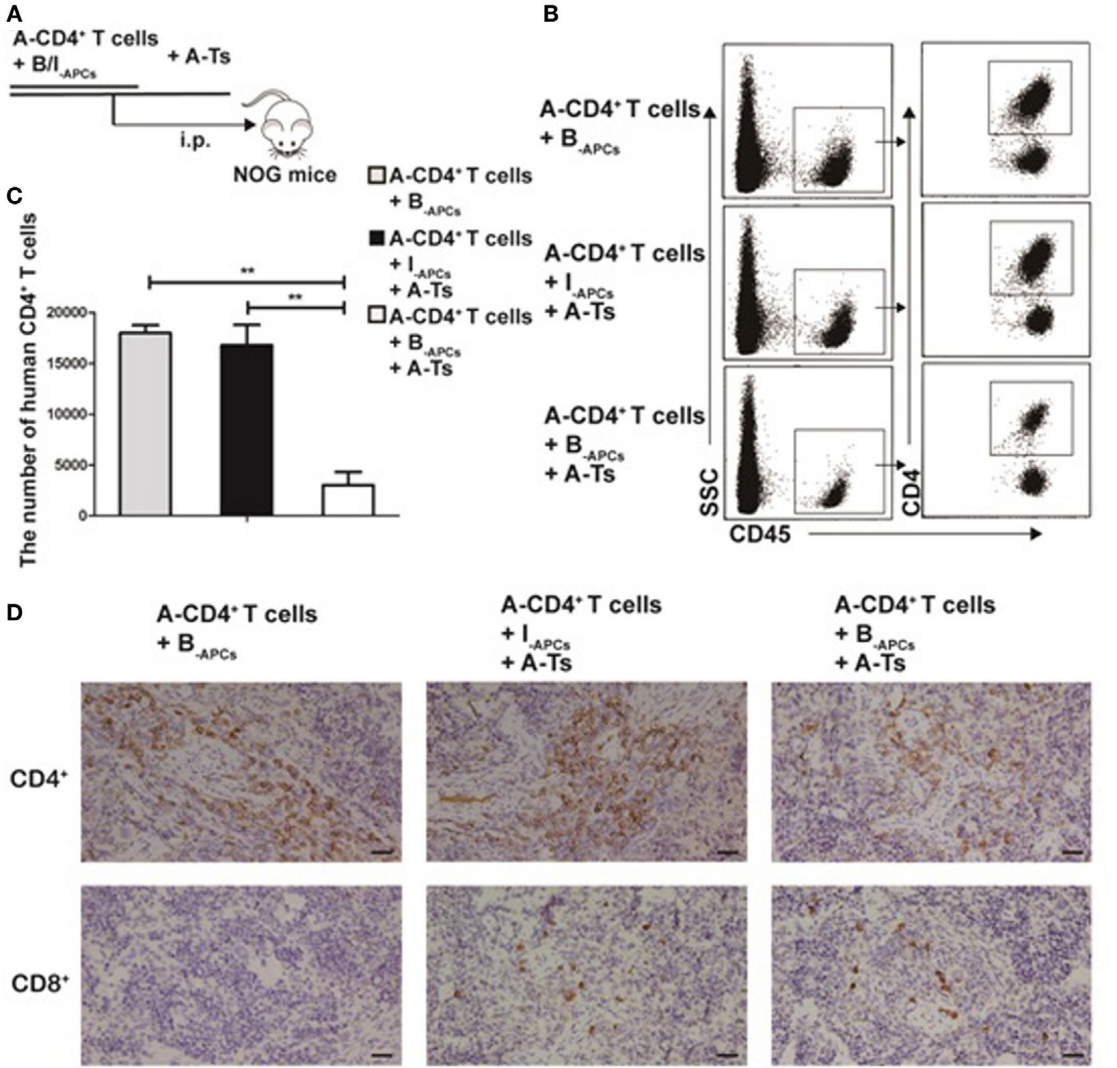
CD8^+^CD28^−^ T cells expanded by IL-15 inhibit allospecific CD4^+^ T cell proliferation *in vivo*. **(A)** 4 × 10^6^ purified responder CD4^+^ T cells (R) from donor A and 4 × 10^6^ antigen-presenting cells (APCs) from donor B (B_-APCs_) or from an indifferent donor I [I_-APCs_, human leukocyte antigen (HLA)-A, -B, and -DR fully mismatched with donor B] were adoptive transferred into NOG mice *via* i.p., the *in vitro* expanded CD8^+^CD28^−^ T cells were added as putative suppressors (S) at S:R ratios of 0.5:1. **(B)** The mice were sacrificed to get splenic suspension on day 11, human CD45 and CD4 were as gates in flow cytometry to assess CD4^+^ T cells number. **(C)** The bar graphs indicate human CD4^+^ T cell number in mice splenic suspension, which were average value for three independent experiments. **(D)** Immunohistochemical staining in NOG mouse spleen tissues (magnification, 400×) showed human CD4^+^ T cell (A-CD4^+^ T cells) and CD8^+^ T cells (human CD8^+^CD28^−^ T cells, A-Ts). Representative data of three mice in three independent experiments. The bar graphs indicate the means ± SD. *p < 0.05, **p < 0.01, and ***p < 0.001.

To investigate whether the CD8^+^CD28^−^ Ts driven by IL-15 could steadily exert allospecific immunosuppressive function *in vivo*, we used an adoptive transfer system in NOG mice. A-CD4^+^ T cells (4 × 10^6^), B_-APCs_ (4 × 10^6^), or I_-APCs_ (4 × 10^6^) and A-Ts CD8^+^CD28^−^ T cells (2 × 10^6^) generated as above were adoptively transferred into NOG mice *via* intraperitoneal injection (Figure 4A). The mice were sacrificed on day 11 and the spleen was harvested and analyzed for total CD4^+^ T cell numbers. Human CD45 and CD4 were used to gate out human CD4^+^ T cells in FACS analysis. As shown in Figures 4B,C, a significantly lower number of CD4^+^ T cells was observed when both stimulator B_-APCs_ and A-Ts suppressor CD8^+^CD28^−^ T cells were transferred, compared with when only stimulator B_-APCs_ were transferred in the absence of A-Ts cells (*p* < 0.001). In contrast, when I_-APCs_ instead of B_-APCs_ was transferred as the stimulator, the number of A-CD4^+^ T cells did not change much (Figures 4B,C), which indicated that the suppression by A-Ts CD8^+^CD28^−^ T cells was donor-specific. As shown in Figure 4D, the immunohistochemistry data of mice spleen further supported the above FACS findings, i.e., in the presence of A-Ts CD8^+^CD28^−^ T cells, the number of A-CD4^+^ T cells stimulated by B_-APCs_ was profoundly suppressed. However, suppression was almost lost when I_-APCs_ was used as stimulator even in the presence of CD8^+^CD28^−^ T cells. Furthermore, as shown in Figure 4D, on day 11 post adoptive transfer, human CD8^+^ cells could be readily found in the spleen, suggesting that the adoptively transferred post-expansion CD8^+^CD28^−^ Ts cells remained viable. Taken together, these findings indicate that the *in vitro* expanded CD8^+^CD28^−^ Ts cells steadily exert allospecific immunosuppressive function and remain viability *in vivo*.

### Cytotoxicity Does Not Contribute to the Allospecific Suppression of CD8^+^CD28^−^ T Cells Expanded *In Vitro*

**Figure 5:**
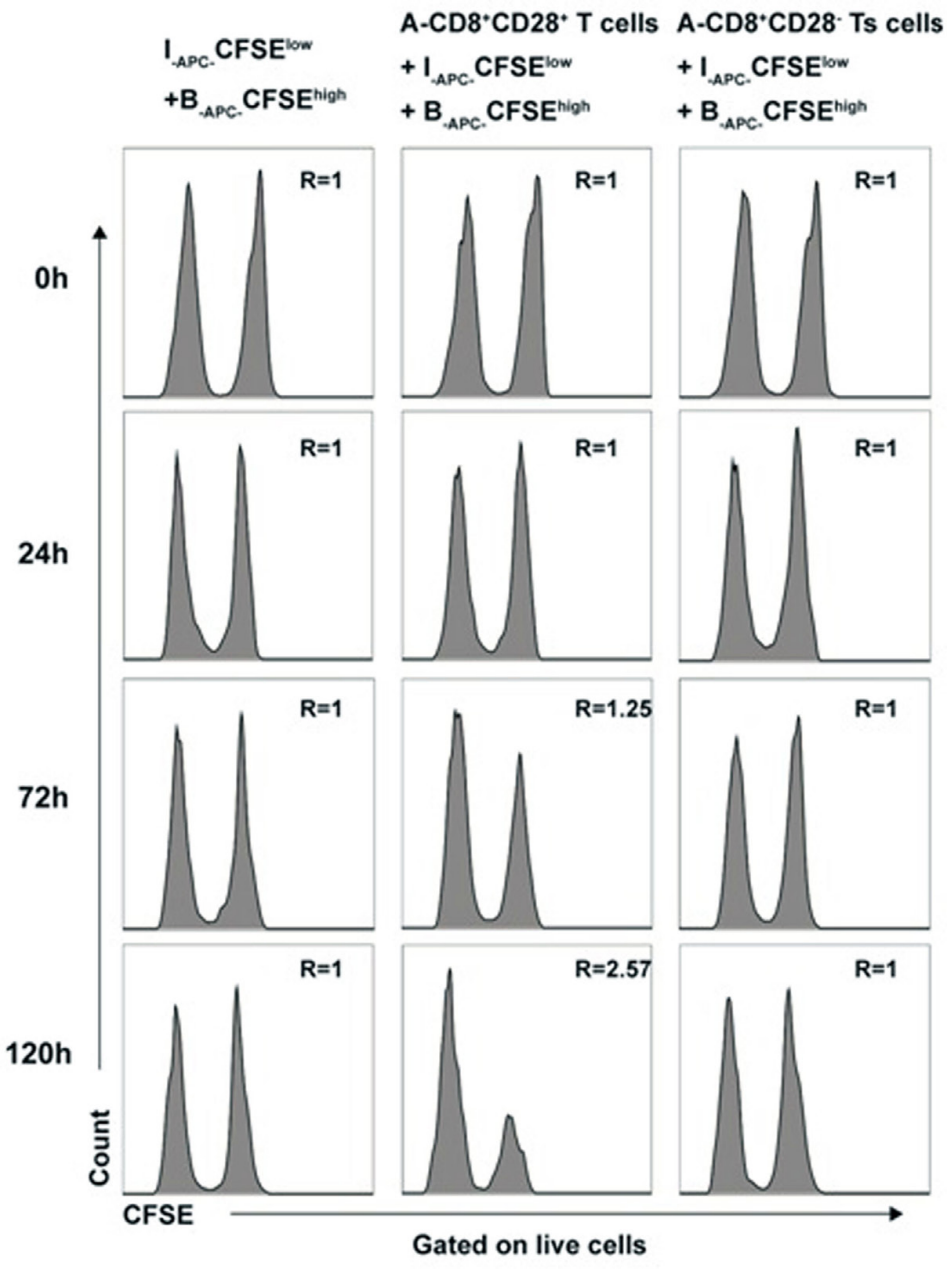
Cytotoxicity does not contribute to the allospecific suppression of CD8^+^CD28^−^ T cells expanded *in vitro* B_-APC-_CFSE^high^ [priming antigen-presenting cells (APCs) from donor B, were labeled with a high concentration (2.0 µM) of carboxyfluorescein diacetate succinimidyl ester (CFSE)] and I_-APC-_CFSE^low^ [APCs from indifferent individual I, labeled with a low concentration (0.2 µM) of CFSE] were cocultured in a mixture of 1:1 by themselves or either with the same number of CD8^+^CD28^+^ T cells (middle panels) or CD8^+^CD28^−^ Ts cells (right panels) in 96 *U*-bottom plates in triplicates. At 0, 24, 72, and 120 h of culture, cells were collected and analyzed by FACS. The ratio of cell numbers of I_-APC-_CFSE^low^ vs B_-APC-_CFSE^high^ was calculated as “*R*” for all time points. An increase in the value of *R* indicates specific killing of B_-APC-_CFSE^high^ cells by the putative effctor cells, whereas a stable value of *R* at 1.0 indicates no specific killing of B_-APC-_CFSE^high^ cells. Data shown are representative of three independent experiments.

To rule out that cytotoxicity toward priming stimulator (B-_APCs_) in above experiments contributed to the allospecific suppression of the *in vitro* expanded CD8^+^CD28^−^ T cells, we directly assessed the cytotoxicity of *in vitro* expanded CD8^+^CD28^−^ T cells by using a CFSE-based assay. Briefly, the CD8^+^CD28^−^ T cells (A-Ts) or CD8^+^CD28^+^ T cells were designated as effector cells, B_-APC_ cells labeled with high concentration of CFSE (B-_APC_-CFSE^high^), and I-_APC_ cells labeled with low concentration of CFSE (I-_APC_-CFSE^low^) were used as target cells in MLRs culture system. I-_APC_-CFSE^low^ and B-_APC_-CFSE^high^ cells at a ratio of 1:1 were cultured in the presence of CD8^+^CD28^−^ T cells or CD8^+^CD28^+^ T cells. As shown in Figure 5, at 0, 24, 72, and 120 h time points, the ratio of I-_APC_-CFSE^low^ vs B-_APC_-CFSE^high^ cells remained unchanged in present of CD8^+^CD28^−^ T cells (Figure 5, left and right panel), but in the presence of CD8^+^CD28^+^ T cells, the ratio rose to 1.25 and 2.57 at 72 and 120 h time point, respectively (Figure 5, middle panel). These results indicate that the *in vitro* expanded CD8^+^CD28^−^ T cells did not exert cytotoxicity toward their priming stimulator B_-APC_ cells, but their counterparts CD8^+^CD28^+^ T cells do. Therefore, we conclude that cytotoxicity did not contribute to the allospecific suppression of the *in vitro* expanded CD8^+^CD28^−^ T cells.

### Suppression by the *In Vitro* Expanded CD8^+^CD28^−^ T Cells Is Contact Dependent

**Figure 6:**
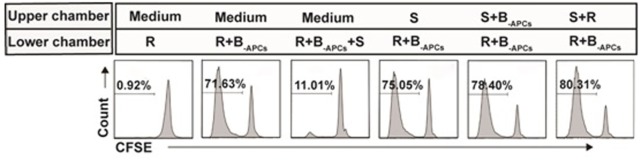
Suppression by the *in vitro* expanded CD8^+^CD28^−^ T cells is contact dependent. Transwell assays: the lower chambers of 96-well transwell plates were plated with carboxyfluorescein diacetate succinimidyl ester (CFSE)-labeled responder cells (5 × 10^4^ of CD4^+^ T cells, R) and stimulator cells (5 × 10^4^ of B_-APCs_), the *in vitro* expanded CD8^+^CD28^−^ T cells (2.5 × 10^4^, S) were added either in the lower chamber to allow cell–cell contact or in the upper chamber to prevent cell–cell contact. The proliferation of the CD4^+^ T cells was measured by CFSE dilution assay. Data shown were representative of three independent experiments.

Contact dependent had been reported to be one of the mechanisms for CD8^+^CD28^−^ T cells to exert their suppression function (24). Next, we determined whether CD8^+^CD28^−^ T cells expanded in this report also suppressed in a contact-dependent manner. Transwell assays were performed as described in Section “Materials and Methods.” As shown in Figure 6, the lower chamber was plated with CFSE-labeled A-CD4^+^ T cells as responder cells (R) and stimulator cells (B_-APCs_), and the *in vitro* expanded CD8^+^CD28^−^ T cells (S) were added either in the lower chamber to allow cell–cell contact or in the upper chamber to prevent cell–cell contact. The suppression of proliferation by CD8^+^CD28^−^ T cells was abolished when the suppressor cells were plated in the upper chamber of the transwell, either alone or combined with responder cells or stimulator cells (Figure 6), indicating that suppression by these cells required cell–cell contact between the CD8^+^CD28^−^ T cells, stimulator APCs, and/or the responder CD4^+^ T cells.

Cytokines include IL-2, IL-10, TGF-β, and IFN-γ in supernatant of different culture conditions on day 7 were assessed for their possibility to involve in the suppression by CD8^+^CD28^−^ Ts cells. As show in Figure S1 in Supplementary Material, the level of IL-2, IL-10, and TGF-β do not have significant differences whether in the presence or absence of CD8^+^CD28^−^ Ts cells, the results suggest that IL-10 and TGF-β do not play a role in the suppression by CD8^+^CD28^−^ Ts cells. At the same time, the level of IFN-γ was found to be significantly lower when the proliferation of CD4^+^ T cells was suppressed by CD8^+^CD28^−^ Ts cells. The reasonable interpretation of lower level of IFN-γ in the supernatant should be the suppressed proliferation of CD4^+^ T cells rather than others. To sum up, suppression by the *in vitro* expanded CD8^+^CD28^−^ T cells is contact dependent but IL-10, TGF-β, and IFN-γ dependent.

### Phenotypic Characteristics of the *In Vitro* Expanded CD8^+^CD28^−^ T Cells

**Figure 7:**
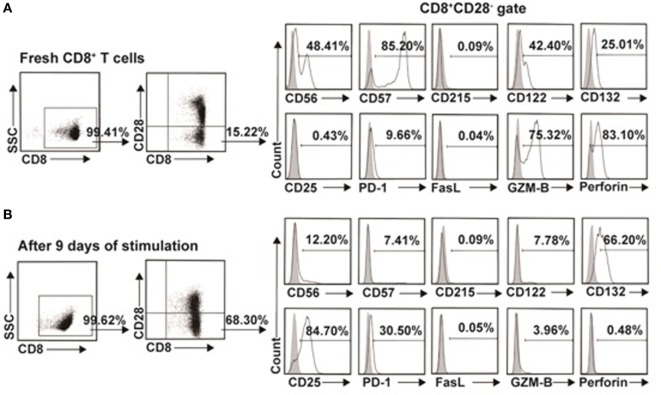
Phenotypic characteristics of freshly isolated and *in vitro* expanded CD8^+^CD28^−^ T cells. **(A)** CD8^+^CD28^−^ T cells freshly isolated from healthy volunteers or **(B)** *in vitro* expanded by IL-15 plus donor antigen-presenting cellss for 9 days were analyzed for cell surface or intracellular markers by multichromatic flow cytometry. Data shown were representative of three independent experiments.

To further investigate the difference between the *in vitro* expanded CD8^+^CD28^−^ T cells and CD8^+^CD28^−^ T cells freshly isolated from PBMC, we examined the phenotypic characteristic of these two populations. As shown in Figure 7A as representative of three independent experiments, freshly isolated CD8^+^CD28^−^ T cells expressed high levels of CD56 (48.85 ± 0.64%), CD57 (86.35 ± 1.63%), CD122 (40.15 ± 3.18%), GZM-B (73.00 ± 3.25%), and perforin (79.25 ± 5.44%), and with minimal expression of CD215 (0.10 ± 0.02%), CD25 (0.67 ± 0.33%), PD-1 (10.30 ± 1.58%), and FasL (0.04 ± 0.01%). In contrast, the *in vitro* expanded CD8^+^CD28^−^ T cells significantly downregulated their expression of CD56 (12.30 ± 0.14%), CD57 (7.01 ± 0.57%), CD122 (7.69 ± 0.13%), GZM-B *(*4.33 ± 0.52%), and perforin (0.44 ± 0.06%), whereas significantly upregulated CD132 (68.20 ± 2.83%), CD25 (81.50 ± 4.53%), and PD-1 (32.63 ± 1.87%) (Figure 7B). Although the exact percentage changes varied in CD8^+^CD28^−^ T cells in different individuals, the trend of up- or downregulation of these markers remained the same in three independent experiments.

### PD-1:PD-L1 Signaling Plays a Critical Role in the Suppressive Function of *In Vitro* Expanded CD8^+^CD28^−^ Ts Cells

**Figure 8:**
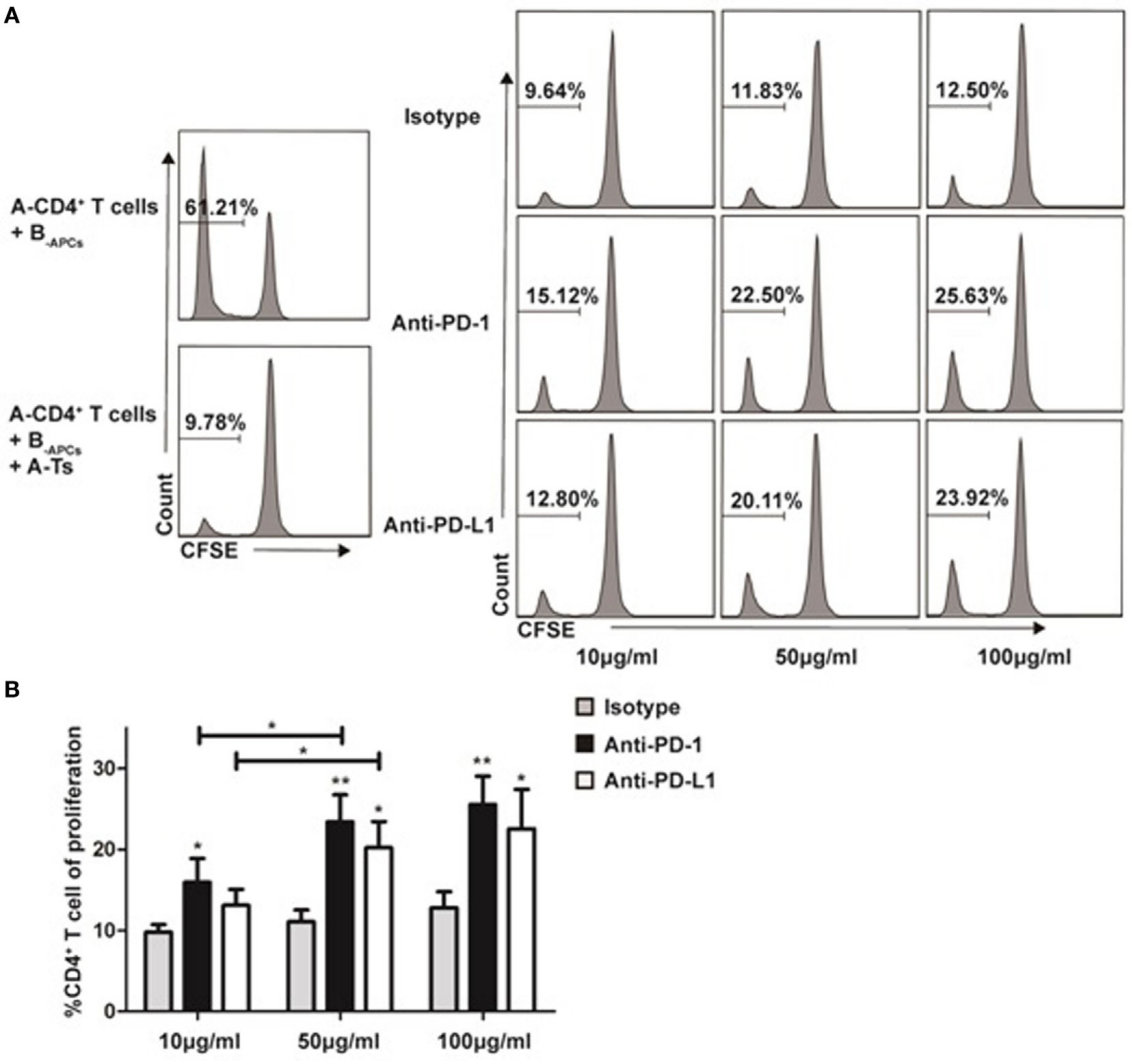
Programmed death-ligand 1 (PD-1):programmed death-ligand 1 (PD-L1) signaling plays a critical role in the suppressive function of *in vitro* expanded CD8^+^CD28^−^ Ts cells Suppression assays were set up as described in Figure 3A at an S:R ratio of 0.5:1. The proliferation of the CD4^+^ T cells was measured by carboxyfluorescein diacetate succinimidyl ester dilution. **(A)** The percentages of proliferating CD4^+^ T cells in the presence of anti-human PD-1, anti-human PD-L1 or isotype control antibody at concentrations of 10, 50, or 100 µg/ml were shown. **(B)** The average percentages of proliferating CD4^+^ T cells in different antibody concentration groups. The bar graphs indicate the means ± SD, *n* = 3. * and ** indicate significant difference within group and between different groups. *p < 0.05 and **p < 0.01.

Recently, co-inhibitory molecule PD-1 and its ligand (PD-L1) were found to play an important role in the maintenance of peripheral tolerance in autoimmune diseases (25) and transplantation (26). As show in Figure 7, we found that the *in vitro* expanded CD8^+^CD28^−^ T cells significantly upregulated PD-1 expression when compared with freshly isolated cells. Thus, we questioned whether PD-1:PD-L1 pathway was implicated in the suppression of CD8^+^CD28^−^ Ts cells. Anti-PD-1 or anti-PD-L1 antibodies were added to the suppression assays described above (baseline suppression without the blocking antibodies was shown in Figure 8A, right top panel). As shown in Figures 8A,B, administration of anti-PD-1 at concentration of 10, 50, or 100 µg/ml significantly impaired the suppressive capacity of CD8^+^CD28^−^ Ts cells as compared with the isotype control (*p* = 0.026, *p* = 0.004, and *p* = 0.005, respectively). Similarly, administration of anti-PD-L1 at concentration of 50 or 100 µg/ml could also impaired the suppressive capacity of Ts cells significantly (*p* = 0.011 and *p* = 0.032, respectively). Taken together, these findings suggested that blockage of PD-1:PD-L1 pathway significantly dampened the suppressive capacity of *in vitro* expanded CD8^+^CD28^−^ Ts cells.

## Discussion

CD8^+^CD28^−^ T suppressor cells have emerged as an important modulator of alloimmunity and autoimmunity and have been reported to be generated *in vitro* after multiple rounds of stimulation of human PBMC with either allogeneic- (27) or xenogeneic-donor APCs (28). Similarly, CD8^+^CD28^−^ Ts can be generated *in vitro* by priming PBMCs with self-APCs pulsed with nominal antigens such as MHC antigens (29) or recombinant tetanus toxoid (30). These approaches, however, demonstrated limited capacity for expansion of CD8^+^CD28^−^ Ts cell numbers *in vitro*, therefore, having limited utility for further research on the characteristics of CD8^+^CD28^−^ T suppressor cells or for clinical applications in the future.

Our previous study reported a novel strategy to expand large numbers of human CD8^+^CD28^−^ Ts cells *in vitro* by stimulating human CD8^+^ T cells with allogeneic APCs supplemented with cytokines cocktail including IL-2, IL-7, and IL-15 (24). In this study, we optimized the expansion condition by using IL-15 alone instead of cytokines cocktail. We found that IL-15 could promote rapid expansion of CD8^+^CD28^−^ T cells in a short period of time like the cytokines cocktail did. Furthermore, we found that IL-15 promotes the expansion of CD8^+^CD28^−^ T cells through different mechanisms, which include promoting the proliferation of CD8^+^CD28^−^ T cells, inducing conversion of CD8^+^CD28^+^ T cells to CD8^+^CD28^−^ T cells, and decreasing the apoptosis of proliferating CD8^+^CD28^−^ T cells.

CD8^+^CD28^−^ T cells freshly isolated from PBMCs account for only a small fraction in CD8^+^ T cells and are also polyclonal. During the IL-15 stimulated expansion, allospecific clones are selectively expanded by the presence of B_-APC_, conferring allospecificity to the resulting CD8^+^CD28^−^ Ts cells. Therefore, it is expected that in comparison to allo-APC expanded CD8^+^CD28^−^ T cells, freshly isolated CD8^+^CD28^−^ T cells are significantly less robust in suppressing allo-stimulated CD4^+^ T cell proliferation.

Our previous work demonstrated the donor-specific suppressive capacity *in vitro* of the expanded CD8^+^CD28^−^ Ts cells in condition of cytokines cocktail include IL-2, IL-7, and IL-15 (24), we testified in this study that IL-15 expanded CD8^+^CD28^−^ Ts cells have similar donor-specific suppressive capacity *in vitro*. Most importantly, we further confirmed the stability of the suppressive capacity *in vivo* circumstances when the expanded CD8^+^CD28^−^ T cells were adoptive transferred into NOG mice. Staining for human CD8^+^ T cells in the spleen of the NOG mice by immunohistochemistry confirmed the sustained viability of these adoptively transferred CD8^+^CD28^−^ Ts cells. To our knowledge, this is the first report to demonstrate the *in vitro* expanded human CD8^+^CD28^−^ T cells exert allospecific suppression *in vivo*.

Although suppressive characteristic of CD8^+^CD28^−^ Ts cells have been reported (19, 20, 24), there are other studies, which implies their cytotoxicity (31). In this study, we set up a CFSE-based assay as our previous report (24) and ruled out the cytotoxicity of CD8^+^CD28^−^ Ts cells toward the priming APCs, which might contribute to the donor-specific suppression of proliferation. The results indicated that the CD8^+^CD28^−^ T cells exhibit no specific cytolysis toward the priming APCs whereas CD8^+^CD28^+^ T cells do have cytotoxicity toward the priming APCs.

*In vitro* expanded CD8^+^CD28^−^ T suppressor cells described in this report were obviously a different population from that of freshly isolated from human PBMCs. As shown in Figure 7, we found that *in vitro* expanded CD8^+^CD28^−^ Ts cells upregulated CD132 (IL-15Rγ), downregulated CD122 (IL-2R/IL-15Rβ), whereas CD215 (IL-15Rα) did not change much when compared with freshly isolated ones, since IL-15 might bind to IL-15Rα in complex with the IL-2R/IL-15Rβ and the common gamma (γc) chain IL-15Rγ to deliver proliferative signaling to CD8 T cells, which might hint that IL-15 promote rapid expansion of CD8^+^CD28^−^ T cells by binding with IL-15Rγ. Upregulation of CD25 in expanded CD8^+^CD28^−^ T suppressor cells was in line with their active state. The study also shows that the expanded CD8^+^CD28^−^ Ts cells downregulated CD56^+^ and CD57^+^, two of the markers of induced CD8^+^ Tregs. This finding suggested that CD8^+^CD28^−^ Ts cells were different from CD8^+^CD56^+^ Tregs and CD8^+^CD57^+^ Tregs.

Several studies in rodents and humans had described the killing of the target cell by CD8^+^ Tregs *in vitro* (32–34), and GZM-B, perforin, and FasL had been considered to be the effector molecules involved in the killing (35–37). However, our results showed that *in vitro* expanded CD8^+^CD28^−^ Ts cells downregulated GZM-B and perforin expression, FasL expression remained in low level. These results were, therefore, consistent with the observed lack of cytotoxicity.

Most interestingly, we found that co-inhibitory molecules PD-1 were upregulated when compared with freshly isolated ones from human PBMC (32.63 ± 1.87 vs 10.30 ± 1.58%, *p* < 0.001) (data not shown). Moreover, suppression by CD8^+^CD28^−^ Ts cells was partially abolished when anti-PD-1 or anti-PD-L1 was added into the suppressing assay (Figure 8). Co-inhibitory signals PD-1:PD-L1 pathway had been found to play a key role in T cell exhaustion in chronic viral infection diseases (38), autoimmunity (25, 39), antitumor immunity (40), and recently in allograft tolerance in animal transplant model (41). Our results indicate that PD-1:PD-L1 pathway plays a role in the suppression mechanisms for CD8^+^CD28^−^ Ts cells although it does not account for all. Since co-inhibitory signals include a series of molecules, such as PD-1, TIM-3, LAG-3, CTLA-4, and their ligands (42, 43), ongoing work in our lab attempts to clarify their function, which will further illuminate the suppression mechanisms of *in vitro* expanded CD8^+^CD28^−^ Ts cells.

In conclusion, our results indicate that IL-15 plus donor APCs could induce rapid expansion of human allospecific CD8^+^CD28^−^ Ts cells; these cells could steadily exert their immunosuppressive function both *in vitro* and *in vivo* circumstances. Co-inhibitory signals *via* the PD-1:PD-L1 pathway plays a role in their suppression mechanisms. These findings may facilitate the understanding of transplant rejection and promote clinical application of CD8^+^CD28^−^ Ts cells-based strategies for inducing and monitoring transplant tolerance in the future.

## Ethics Statement

All of the experiments using human cells were carried out in accordance with the recommendations of the ethics committee of Southern Medical University (Guangzhou, China); all subjects gave written informed consent in accordance with the Declaration of Helsinki. All of the animal experiments were approved by the institutional animal care and use committee of Southern Medical University and were performed in accordance with guidelines set forth by Southern Medical University.

## Author Contributions

Conceived and designed the experiments: YY and YL. Performed the experiments: FF, YL, GL, PZ, MZ, and HZ. Analyzed the data: YY, YL, JL, and XL. Contributed reagents/materials/analysis tools: YY, YL, and XL. Wrote the paper: FF and YY.

## Conflict of Interest Statement

The authors declare that the research was conducted in the absence of any commercial or financial relationships that could be construed as a potential conflict of interest.

## Abbreviations

APCs, antigen-presenting cells; Tregs, regulatory T cells; Ts, T suppressor; γc, γ chain; PBMC, peripheral blood mononuclear cell; HLA, human leukocyte antigen; MLR, mixed lymphocyte reaction; Abs, antibodies; CD, cluster of differentiation; CFSE, carboxyfluorescein diacetate succinimidyl ester; Foxp3, forkhead box P3; PD-1, programmed cell death protein 1; PD-L1, programmed death-ligand 1; GZM-B, granzyme B; FasL, Fas ligand.
