# Supplementary material for: Human CD8+CD28− T Suppressor Cells Expanded by IL-15 In Vitro Suppress in an Allospecific and Programmed Cell Death Protein 1-Dependent Manner
Source: Front Immunol. 2018 Jun 22;9:1442. doi: 10.3389/fimmu.2018.01442 (PMC6023977; doi:10.3389/fimmu.2018.01442)
Supplement: Supplementary file 1 [file Data_Sheet_1.docx]

Supplementary Material

**Human CD8^+^CD28^-^ T Suppressor Cells Expanded by IL-15 *In Vitro* Suppress in an Allospecific and PD-1-dependent Manner**

**Fu Feng^1, 2§^, Yanjun Liu^3§^, Guihuan Liu^3^, Ping Zhu^3^, Manman Zhu^3^, Hua Zhang^1,2^, Xiao Lu^3^, Jiumin Liu^2^, Xunrong Luo^4^, Yuming Yu^2, 1*^**

**^§^**Fu Feng and Yanjun Liu are co-first authors.

**^*^** Correspondence: Yuming Yu: [yuym72@163.com](mailto:yuym72@163.com)

**Supplementary Figures**


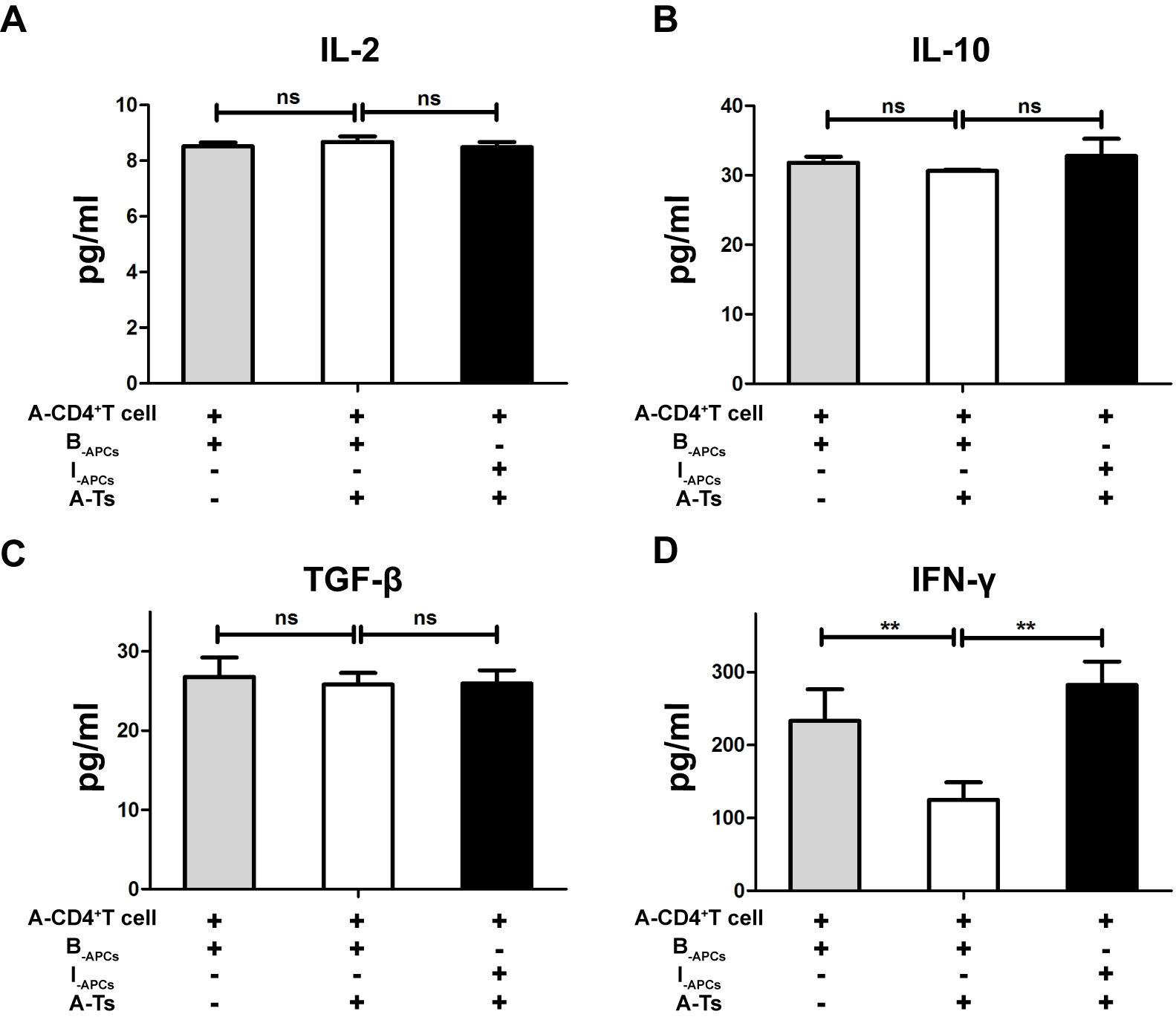


**Supplementary Figure 1. Suppression by the in vitro expanded CD8^+^CD28^-^ T cells is IL-10 or TGF-β independent.**

The supernatant was harvested on day 7 and the concentration of cytokines (i.e. IL-2, IL-10, TGF-β and IFN-γ) under three different culture conditions was analyzed. Significant differences were calculated and shown (ns: no significant, * P < 0.05, ** P < 0.01).
